# Correction: Cationic conjugated polymer-based FRET aptasensor for label-free and ultrasensitive ractopamine detection

**DOI:** 10.1039/d2ra90045a

**Published:** 2022-04-22

**Authors:** Xuancheng Fu, Wei Dong, Chang Liu, Jing Han, Chengzhi Huang

**Affiliations:** School of Sport Science, Beijing Sport University Beijing 100084 China; Institute of Anti-Doping in China, Beijing Sport University Beijing 100084 China; Beijing National Laboratory for Molecular Sciences, Institute of Chemistry Chinese Academy of Sciences Beijing 100190 P. R. China jinghan@iccas.ac.cn; Key Laboratory of Luminescent and Real-Time Analytical Chemistry (Southwest University), Ministry of Education, College of Pharmaceutical Sciences, Southwest University Chongqing 400715 China chengzhi@swu.edu.cn

## Abstract

Correction for ‘Cationic conjugated polymer-based FRET aptasensor for label-free and ultrasensitive ractopamine detection’ by Xuancheng Fu *et al.*, *RSC Adv.*, 2022, **12**, 10911–10914, https://doi.org/10.1039/d2ra00574c.

The authors regret that an asterisk was not displayed next to the name of the corresponding author Chang Liu in the original manuscript. The corrected information is shown above.

The Royal Society of Chemistry apologises for these errors and any consequent inconvenience to authors and readers.

## Supplementary Material

